# Development of immunocompatible pluripotent stem cells via CRISPR-based human leukocyte antigen engineering

**DOI:** 10.1038/s12276-018-0190-2

**Published:** 2019-01-07

**Authors:** Yeonsue Jang, Jinhyeok Choi, Narae Park, Jaewoo Kang, Myungshin Kim, Yonggoo Kim, Ji Hyeon Ju

**Affiliations:** 1 Catholic iPSC Research Center, College of Medicine, The Catholic University of Korea, Seoul, 137-701 South Korea; 2 Convergent Research Consortium for Immunologic Disease, Seoul St. Mary’s Hospital, Seoul, 137-701 South Korea; 3 Department of Laboratory Medicine, College of Medicine, The Catholic University of Korea, Seoul, 137-701 South Korea; 4 Catholic Genetic Laboratory Center, Seoul St. Mary’s Hospital, College of Medicine, The Catholic University of Korea, Seoul, 137-701 South Korea; 5 Division of Rheumatology, Department of Internal Medicine, Seoul St. Mary’s Hospital, College of Medicine, The Catholic University of Korea, Seoul, 137-701 South Korea

**Keywords:** Induced pluripotent stem cells, Genetic engineering, Allotransplantation

## Abstract

Pluripotent stem cell transplantation is a promising regenerative strategy for treating intractable diseases. However, securing human leukocyte antigen (HLA)-matched donor stem cells is extremely difficult. The traditional approach for generating such cells is to establish homozygous pluripotent stem cell lines. Unfortunately, because of HLA diversity, this strategy is too time-consuming to be of practical use. HLA engineering of donor stem cells has been proposed recently as a means to evade graft-versus-host rejection in stem cell allotransplantation. This approach would be advantageous in both time and cost to the traditional method, but its feasibility must be investigated. In this study, we used CRISPR/Cas9 to knockout HLA-B from inducible pluripotent stem cells (iPSCs) with heterogenous HLA-B and showed that the HLA-B knockout iPSCs resulted in less immunogenicity in HLA-B antisera than that in the control. Our results support the feasibility of HLA-engineered iPSCs in stem cell allotransplantation.

## Regenerative medicine: Engineering compatible stem cells

Blocking the expression of genes that regulate the immune response in therapeutic stem cells could increase the chances of success following transplantation. Discrepancies between human leukocyte antigen (HLA) genes in a patient and those in transplanted stem cells can cause a damaging immune response and transplantation failure, yet matching HLA types between donors and recipients is notoriously difficult. Ji Hyeon Ju at The Catholic University of Korea in Seoul and colleagues have used the CRISPR/Cas9 gene editing system to introduce a mutation in the HLA-B gene that prevents its expression in pluripotent stem cells derived from adult cells. These modified cells not only retain their capacity to self-renew and differentiate, they are also less likely to trigger an immune response. This promising new approach could reduce the time and cost of developing effective stem cell therapies.

## Introduction

Stem cell therapy is a promising regenerative medicine approach for treating patients with intractable diseases. For stem cell therapy to be successful, at least two criteria must be met before transplantation: the donor and recipient must be HLA-matched, and a sufficient number of donor stem cells must be secured^1–3^. To date, mesenchymal stem cells (MSCs) have been the most widely used source of stem cells^4^, but it is difficult to acquire sufficient numbers of these cells for transplantation^5–7^. Recently, inducible pluripotent stem cells (iPSCs) have emerged as an alternative source of MSCs; these cells are both highly expandable and reproducible^8–10^, enabling the preparation of sufficient numbers of donor stem cells in advance of transplantation. Nevertheless, HLA-matched donor stem cells are still difficult to secure. HLA engineering of donor stem cells has been proposed as a potential solution to this problem, but its feasibility has not yet been demonstrated in human iPSCs^11^. In this study, we developed immunocompatible, ready-to-use donor stem cells and then tested their suitability for transplantation availability by HLA-targeted complement-dependent cytotoxicity assays.

iPSCs, which are generated from mature somatic cells, have differentiation capabilities similar to those of embryonic stem cells (ESCs)^12,13^. Unlike MSCs, iPSCs maintain cell proliferation and differentiation ability across generations, one of the most important properties for a stem cell source. In addition, iPSCs can be genetically manipulated to produce customized stem cells^14,15^. Unfortunately, however, the development of iPSCs is costly and time-consuming, which represents a critical obstacle to the development of patient-specific iPSCs^16,17^. As donor stem cells for the treatment of an inherited disease, normal stem cells (flawless stem cells from a genetically normal individual) are theoretically preferable to iPSCs, but this material would run the risk of allogeneic transplant rejection^18,19^. The success of allogeneic transplantation depends on the extent of matching HLA alleles between the donor and recipient^20–22^.

The HLA locus encodes the major histocompatibility complex (MHC) proteins responsible for the regulation of the adaptive immunity system in humans^23^. MHC proteins present antigens to immune cells such as cytotoxic T lymphocytes, leading to targeted killing of infected cells. HLA mismatch between the donor and recipient is a major cause of failure of cell or tissue transplantation. In severe cases of HLA mismatch, transplanted immune cells attack host cells, a situation known as graft-versus-host disease (GvHD). Therefore, to guarantee the success of transplantation, HLA-matched stem cells must be provided to the recipient^24–26^. However, matching HLA types between a donor and a recipient is very difficult. According to a previous report, only 25% of the HLA genotype is identical, even between siblings^27^.

In this study, to increase immunocompatibility, we knocked out heterozygous HLA-B from an iPSC (homozygous HLA-A, heterozygous HLA-B) using the CRISPR/Cas9 system^28,29^, which resulted in homozygous HLA-A iPSCs without HLA-B. The HLA-B knockout iPSCs did not express HLA-B but maintained a level of stem cell marker expression similar to that of control cells. In addition, HLA-B knockout iPSCs exhibited less immunogenicity than that in the controls in HLA-targeted complement-dependent cytotoxicity assays. Our results suggest that HLA-modified iPSCs represent a promising source of immunocompatible and ready-to-use donor stem cells to treat human disease.

## Materials and methods

### Establishment of inducible pluripotent stem cells

Cord blood mononuclear cells (CBMCs) were directly obtained from the Cord Blood Bank of Seoul St. Mary’s Hospital. The Institutional Review Board (IRB) of the Catholic University of Korea, Seoul St. Mary’s Hospital approved this study. The reprogramming of CBMCs into iPSCs was induced using the Cytotune-iPS Sendai Reprogramming Kit (Life Technologies, Carlsbad, CA, USA). Briefly, CBMCs were seeded in a 24-well plate (3 × 10^5^ cells/well) with StemSpan™ medium (STEMCELL Technologies, Seattle, WA, USA). After addition of the viral components, the plate was centrifuged at 1160×*g* at 25 °C for 30 min and then incubated at 37 °C in 5% CO_2_. On the next day, the cells were transferred to a 12-well plate coated with vitronectin (Life Technologies). The plate was centrifuged at 1160×g at 25 °C for 10 min, and Essential 8™ medium (Thermo Fisher Scientific, Waltham, MA, USA) was added at a 1:1 ratio. The cells were maintained in E8 medium until iPSC colonies were generated. The colonies were maintained in E8 medium (Thermo Fisher Scientific) on vitronectin-coated culture dishes. iPSCs were passaged every 3–4 days using Accutase Cell Detachment Solution (Global Cell Solutions, North Garden, VA) with Y-27632 dihydrochloride (R&D Systems, Minneapolis, MN, 10 µM). The medium was changed every day.

### Genetic engineering of the *HLA-B* gene

**Fig. 1:**
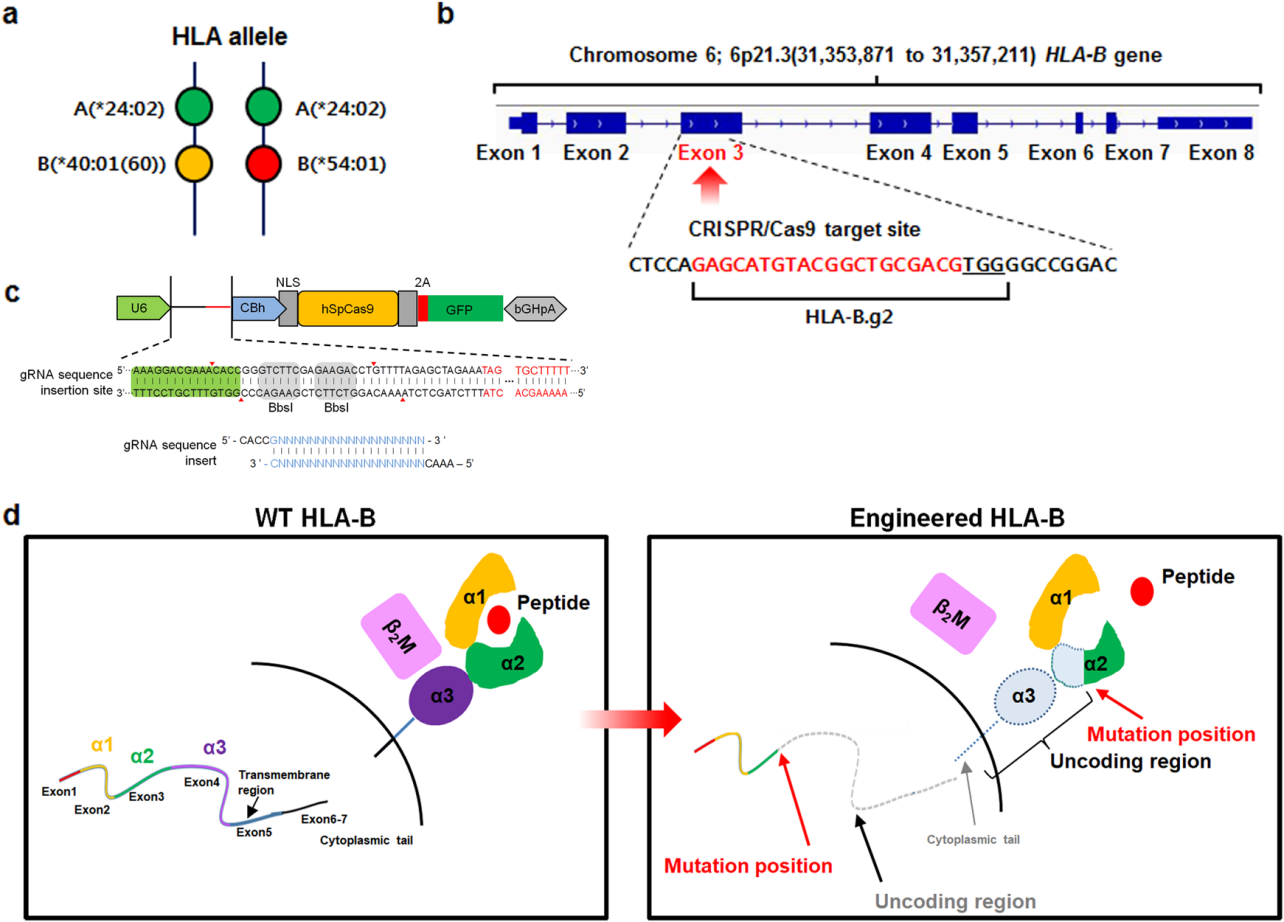
A strategy for developing immunocompatible stem cells using the CRISPR/Cas9 system. **a** HLA allelic type of iPSCs used for immunocompatible cell development. **b** gRNA-targeted gene locus and sequence for HLA-B engineering. HLA-B.g2 (gRNA) was designed to target an exon of the *HLA-B* gene. **c** The vector map of the HLA-B.g2 plasmid. **d** Predicted region of the HLA-B protein deleted by the CRISPR/Cas9 system

The guide RNAs (gRNAs) were designed using the gRNA design tool provided by Applied Stem Cell (Milpitas, CA, USA). Based on the proximity to the target site and off-target profile, the gRNA HLA-B.g2 (5′-GAGCATGTACGGCTGCGACGTGG-3′) was selected. In an in silico off-target analysis of gRNA HLA-B.g2, off-targets with up to two mismatches were not predicted in the coding regions of the human genome (Supplementary Table S1). HLA-B.g2 was cloned into the expression vector pBT-U6-Cas9–2A-GFP (Fig. 1c), and then the resulting plasmid was transfected into iPSCs. To establish an HLA-B-engineered iPSC clone, parental iPSCs (5 × 10^5^) were plated on six-well plates, transfected with the resulting plasmid by electroporation using 1100 V, 30 ms, and 1 P in the Neon Transfection System (Thermo Fisher Scientific, Waltham, MA, USA), cultured for 48 h, and then selected by puromycin (0.2 μg/ml) for another 48 h (2 days after transfection). Since the selected iPSC population was assumed to have mixed types, it was further subjected to limiting dilution for cloning and genotype analysis. Briefly, genomic DNA was extracted from each iPSC clone and then analyzed by Sanger sequencing to identify the insertion or deletion (indels) generated within the HLA-B gene.

### Complement-dependent cytotoxicity assay

The antibody-dependent cellular cytotoxicity (ADCC) assay was performed using the X-celligence assay (ACEA Bioscience Inc. San Diego, CA, USA) according to the manufacturer’s instructions. Briefly, bMSCs and HLA-B-engineered iMSCs (7 × 10^3^/well) were seeded in an E-plate. After stabilizing, the MSCs were treated with an anti-HLA-B antibody (10 ng/μl), incubated for 15 min (at 37 °C), and then treated with rabbit complement (20%) or not for the control. ADCC was evaluated by real-time monitoring of living cells for 10 h.

### *HLA* typing

Sequence-based typing of HLA-B was performed using AlleleSEQR^®^ kits for high-resolution HLA typing (GenDx, Utrecht, The Netherlands). Serological HLA-B typing was performed using Terasaki HLA-ABC Oriental Tissue Typing Trays (One Lambda, Canoga Park, CA, USA) containing known antisera.

### Short tandem repeat analysis

The short tandem repeat (STR) analysis was performed using multiplex amplification by AmpFlSTR Identifier PCR Amplification (Applied Biosystems, Warrington, UK) according to the manufacturer’s instructions. Amplified PCR products were analyzed by capillary electrophoresis using an ABI 3130xl genetic analyzer (Applied Biosystems, Foster City, CA). GeneMapper ID Software Version 4.1 (Applied Biosystems) was used for automated genotyping.

### Karyotyping

Cells at ~80% confluence were added to Chromosome Resolution Additive (Genial Genetic Solutions, Runcorn, UK) and then treated with Colcemid^®^ (PAA Laboratories GmbH, Pasching, Austria) for 30 min. The cells were harvested, treated with prewarmed hypotonic KCl (0.075 M) solution, and fixed with a 1:3 mixture of acetic acid to methanol. G-banding was performed, and the karyotype was described according to the International System for Human Cytogenetic Nomenclature recommendations (2016).

### Semiquantitative reverse transcription-PCR (RT-PCR)

Total RNA was extracted from cells using TRIzol reagent (Invitrogen, Waltham, MA, USA) and reverse-transcribed using a RevertAid™ First Strand cDNA Synthesis kit (Thermo Fisher Scientific). Reverse transcriptase PCR (RT-PCR) and real-time PCR were performed using a TaKaRa PCR Thermal Cycler Dice^®^ Gradient (Takara Bio Inc., Shiga, Japan) and LightCycler 480 (Roche Life Science, Basel, Switzerland), respectively. Primer sequences are provided in Supplemental Table S2.

### Western blot analysis

Total protein was extracted from cells with lysis buffer as previously reported^30^. Protein lysates (30 μg) were resolved by 10–12% SDS–PAGE and transferred to nitrocellulose (NC) blotting membranes (Amersham^TM^ Protran^TM^, Chicago, IL, USA). After blocking with 5% skim milk for 1 h at room temperature (RT), the NC membranes were incubated overnight with the indicated primary antibodies on a shaker at 4 °C. After extensive washing, the membranes were incubated with secondary peroxidase-linked IgG (1:5,000, Abcam, Cambridge, MA, USA) for 1 h at RT. The immunoreactivity was detected by enhanced chemiluminescence (ECL kit, Amersham Pharmacia Biotech, Piscataway, NJ). The image was obtained using an Amersham Imager 600 (Amersham Biosciences Corp., Little Chalfont, Buckinghamshire, UK)

### Immunofluorescence staining

iPSCs (1 × 10^3^ cells/well) were plated in a vitronectin-coated 12-well plate with Y-27632 dihydrochloride (10 uM/ml). To induce the formation of large iPSC colonies, the cells were expanded for 5–7 days with daily media changes. After expansion, iPSCs were washed with PBS, fixed with 4% paraformaldehyde, permeabilized using 0.1% Triton X-100 (Biosesang, Seoul, Korea) for 10 min, and then blocked for 30 min at RT with PBS containing 2% bovine serum albumin (BSA; Sigma-Aldrich, St. Louis, MO, USA) (PBA). The cells were incubated with primary antibodies for 2 h at RT (manufacturers and dilution factors of primary antibodies are provided in Supplementary Table S3) and then with Alexa Fluor 594- (1/400; Life Technologies) or 488-conjugated secondary antibody (1/400; Life Technologies) for 1 h at RT. The cells were washed and mounted using ProLong Antifade mounting reagent (Thermo Fisher Scientific)^31^. Stained colonies were detected with a fluorescence microscope.

### Teratoma formation assay

All animal procedures were conducted under the Laboratory Animals Welfare Act, the Guide for the Care and Use of Laboratory Animals, and the Guidelines and Policies for Rodent Experimentation provided by the Institutional Animal Care and Use Committee of the College of Medicine of the Catholic University of Korea. This study was approved by the Institutional Review Board of the Catholic University of Korea (CUMC‐2016–0291–02). Immunodeficient nude mice (NOD/CB17-Prkdcscid/J) were purchased from Jackson Laboratories, USA. Wild-type iPSCs and each HLA-B-engineered iPSC clone (1 × 10^6^ cells/clone) were suspended in 30 μl of medium-Matrigel (BD) mixture (DMEM/F12 medium: Matrigel 1:1). The cell mixtures were injected into subcutaneous and testis capsules in 7-week-old NOD mice^32^. Eight weeks after injection, when the size of the teratoma of each iPSC clone reached 15 mm in diameter, the teratomas were fixed with 4% paraformaldehyde and processed for hematoxylin and eosin staining for histological evaluation.

### Chondrogenic differentiation and histological analysis

Chondrogenic differentiation was induced using the spheroid culture method^33^. Briefly, each iPSC clone (2 × 10^6^ cells) was resuspended in aggrewell medium (Stemcell Technologies), plated onto a 100-mm petri dish to form an embryoid body (EB) and incubated at 37 °C in 5% CO_2_ for 6 days. At day 6, EBs were harvested, placed on a dish coated with 0.1% gelatin (Sigma, St. Louis, MO, USA) with DMEM containing 20% fetal bovine albumin (FBS), and then allowed to generate outgrowth cells for 1 week. After 7 days, single outgrowth cells (3 × 10^5^ cells/15 ml tube) were prepared by passing through a 40 μm strainer (Thermo Fisher Scientific) in chondrogenic differentiation medium [CDM; DMEM, 20% knockout serum replacement (Thermo Fisher Scientific), 1 × nonessential amino acids (Thermo Fisher Scientific), 1 mM L-glutamine (Sigma), 1% sodium pyruvate (Sigma), 1% ITS + Premix (BD Biosciences), 10^–7^ M dexamethasone (Sigma), 50 mM ascorbic acid (Sigma), 40 μg/ml L-proline (Sigma) supplemented with 50 ng/ml human bone morphogenetic protein 2 (BMP-2, PeproTech, Rocky Hill, NJ), and 10 ng/ml human transforming growth factor beta (TGF-β3, PeproTech)] and centrifuged at 750×*g* for 5 min to promote spheroid formation. Spheroid pellets were maintained for 30 days. The medium was changed every other day. Chondrogenic pellets were stained as previously reported^34^. Briefly, pellets were fixed with 4% paraformaldehyde and embedded in a paraffin block. Each section (7 μm) was stained with 1% Alcian blue, 0.1% Safranin O solution, or 0.04% Toluidine Blue. Hematoxylin and eosin (H&E) and nuclear fast red solution were used as a counterstain. After staining, the sections were mounted using VectaMount™ Permanent Mounting Medium (Vector Laboratories, Burlingame, CA, USA). Stainings were performed in triplicate. Stained chondrogenic pellets were detected with a bright field microscope.

### Endothelial cell differentiation

iPSCs (1.8 × 10^5^ cells) were seeded in a 12-well plate coated with Matrigel (BD Biosciences, San Jose, CA, USA) containing E8 medium. The next day, the medium was replaced with N2B27 medium [50% neurobasal medium (Life Technologies), 50% DMEM F/12 medium (Life Technologies), 5% N2 supplement (Life Technologies), 10% Supplement B-27 minus Vitamin A (Life Technologies), 0.05% β-mercaptoethanol (Life Technologies), 6 μM CHIR99021 (Cayman), and bone morphogenic protein 4 (BMP4, 25 ng/ml)]. The cells were incubated for another 3 days. On day 4, the N2B27 medium was replaced with Stempro-34 SFM (Thermo Fisher Scientific) containing forskolin (2 nM, Abcam, Milton, Cambridge, UK) and vascular endothelial growth factor (VEGF, 100 ng/ml, R&D Systems). The cells were incubated for another 2 days. On day 6, the medium was replaced with Stempro-34 SFM containing Nutrient Supplement and VEGF (50 ng/ml)^35^. The cells were incubated for another 2 days with daily medium changes. On day 8, the cells were harvested and analyzed on a BD LSRFortessa™ Cell Analyzer System (BD Biosciences). The data were analyzed using FlowJo software (Version 10). All experiments were performed in triplicate.

### Functional evaluation of iPSC-derived endothelial cells (iEC)

1. Real-time PCR analysis of ICAM-1 gene expression in TNF-α treated iPSCs:
  The parental and HLA-B-engineered iECs were treated with 10 ng/ml tumor necrosis factor-α (TNF-α PeproTech) for 16 h. Total RNAs were extracted from those cells and then reverse-transcribed into cDNA using a RevertAid™ First Strand cDNA Synthesis kit (Thermo Fisher Scientific). The resultant cDNAs were used for real-time PCR analysis of ICAM-1 expression.
2. LDL uptake measurement
  :The iECs differentiated from wild-type (parental) and HLA-B-engineered iPSCs were placed on fibronectin-coated glass slides (BD Biosciences). After 24 h, the cells were incubated with acetylated low-density lipoprotein labeled with the fluorescent probe 1,1’-dioctadecyl-3,3,3′,3′-tetramethyl-indocarbocyanine perchlorate (Dil-Ac-LDL, Invitrogen, Oregon, 5 μg/mL) at 37 ℃ for 4 h, washed with PBS, and then fixed with 4% paraformaldehyde for 15 min. After washing with PBS, the cells were stained with 4′,6-diamidino-2-phenylindole (DAPI, 5 μg/ml, Roche Diagnostics) for 10 min. The stained cells were washed with PBS and mounted using ProLong Antifade mounting reagent (Thermo Fisher Scientific). Ac-LDL–incorporating cells were detected using a fluorescence microscope (AXIO imager, M2, Carl Zeiss AB, Sweden).
3. Vascular tube formation
  : Growth factor-reduced Matrigel (BD Biosciences) was added into a 96-well plate (50 μl/well) and allowed to solidify for 30 min at 37 °C. The iECs differentiated from wild-type and HLA-B-engineered iPSCs (1.2 × 10^4^) were resuspended with Stempro-34 SFM containing nutrient supplement medium (100 μl) with 50 ng/ml VEGF165 (PeproTech), plated onto the solidified Matrigel, and then incubated for 12 h at 37 °C. For fluorescent monitoring of tube formation, the cells were incubated with calcein AM (Thermo Fisher Scientific, 2 μg/ml) for 30 min at 37 °C and 5% CO_2_ under protection from the light. After washing with PBS, the cells were observed using a fluorescence microscope (AXIO OBSERVER Z1, Carl Zeiss AB, Sweden).

### Mesenchymal stem cell differentiation

MSC differentiation was performed according to our previous protocol^33^. Briefly, each iPSC clone (2 × 10^6^ cells) was resuspended with aggrewell medium (Stemcell Technologies), plated onto a 100-mm petri dish to form an embryoid body (EB), and incubated at 37 °C in 5% CO_2_ for 10 days. At day 10, the EBs were harvested, placed on a dish coated with 0.1% gelatin (Sigma), and allowed to outgrow in DMEM containing 20% FBS for an additional 10 days. The outgrowth cells were passaged to gelatin-coated (0.1% gelatin, RT for 1 h) tissue culture plates up to passage 4 (P4).

### Flow cytometry

Freshly isolated cells were washed and fixed with 4% paraformaldehyde (PFA). After fixation, the cells were washed in fluorescence-activated cell sorting (FACS) buffer (PBS containing 2% FBS) and stained with APC-conjugated anti-human CD34, PE-conjugated anti-human CD73, PE-Cy7-conjugated anti-human CD105, and PE-Cy7-conjugated anti-human CD144 antibodies as previously described^34^. Anti-IgG-conjugated APC, PE, and PE-Cy7 were used as isotype controls. Manufacturers and dilution ratios (or concentrations) of antibodies are provided in Supplemental Table S2. The stained cells were analyzed on a BD LSRFortessa™ Cell Analyzer Systems (BD Biosciences, South San Francisco, CA, USA). The data were analyzed using FlowJo software (Version 10).

### Statistical analysis

All experiments were performed separately at least three times. Data are presented as the mean ± standard deviation. Statistical analysis was performed using a two-tailed Student’s *t* test and a one-way analysis of variance (ANOVA) with post hoc Bonferroni correction. For all tests, *p* < 0.05 was considered to be statistically significant.

## Results

### A strategy for developing immunocompatible stem cells using the CRISPR/Cas9 system

According to an HLA allele analysis of our cord blood bank (127 cases), the source of our iPSCs, HLA-B alleles exhibited the highest heterozygosity and HLA-A alleles exhibited the lowest (Supplemental Table S4). Therefore, to increase the immunocompatibility of iPSCs, we attempted to knockout HLA-B alleles by genetic engineering. As shown in Fig. 1a, the HLA type we selected was A *24:02, B *40:01(60), and *54:01, which was the most abundant type in our cord blood bank (Fig. 1a). For genetic engineering of HLA-B alleles using the CRISPR/Cas9 system, we designed HLA-B.g2 (CRISPR guide RNA (gRNA)) to target exon 3 of the *HLA-B* gene on the short (p) arm of chromosome 6 (Fig. 1b). The gRNA HLA-B.g2 was cloned into the expression vector pBT-U6-Cas9–2A-GFP (Fig. 1c). As shown in Fig. 1d, the engineered *HLA-B* allele lacks part of the alpha 2 domain and all of the alpha 3 domain, and consequently cannot display MHC HLA-B on the cell surface.

### HLA-B sequence and immunogenicity analysis of CRISPR-engineered inducible pluripotent stem cells

**Fig. 2:**
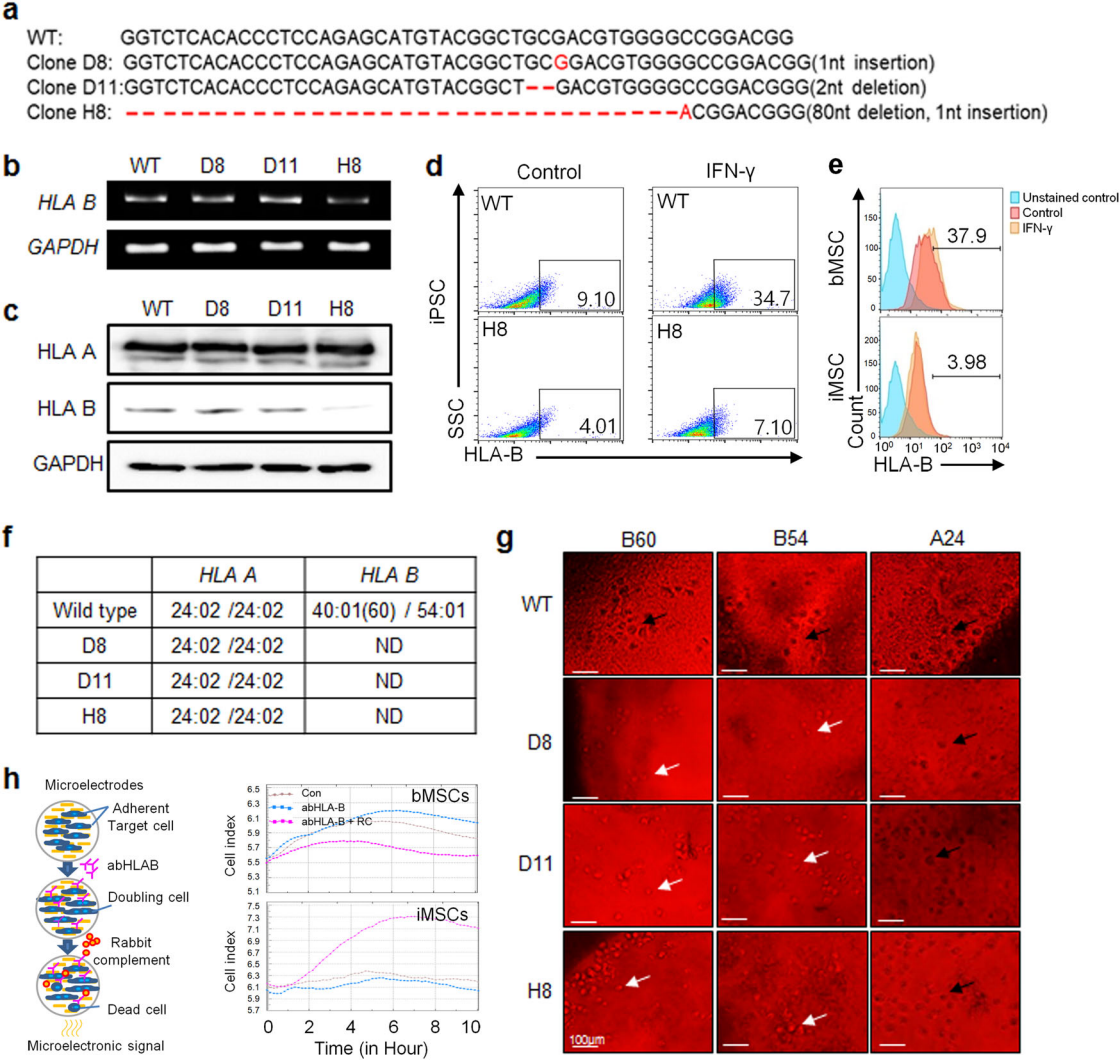
HLA-B sequence and immunogenicity analysis of CRISPR-engineered inducible pluripotent stem cells. **a** Nucleotide sequence analysis of HLA-B–engineered iPSC clones. Expression analysis of HLA-B in HLA-B-engineered iPSC clones at the **b** mRNA and **c** protein levels. The flow cytometric analysis of HLA-B-expression in **d** wild-type and HLA-B-engineered iPSCs and **e** HLA-B-engineered iPSC-derived MSCs. The cells were unstimulated or stimulated with IFN-γ for the induction of HLA-B overexpression. bMSCs were used as the control. **f** HLA-typing analysis of HLA-B-engineered iPSC clones. HLA typing was performed with sequence-based typing (SBT). (ND; not determined). **g** Complement-dependent cytotoxicity analysis of HLA-B-engineered iPSC clones. To analyze complement-dependent cytotoxicity, serological HLA-B typing was performed using Terasaki HLA Tissue Typing Trays. For complement-dependent cytotoxicity, the cells were incubated with HLA–A (*24) or –B (*54 or *60) specific antiserum. Living and dead cells were determined by eosin staining. Black arrows represent dead cells. White arrows indicate live cells. **h** X-celligence scheme (left panel). Antibody-dependent cellular cytotoxicity (ADCC) of HLA-B-engineered iPSC-derived MSCs using x-celligence (right panel). bMSCs were used as the control

Three clones (D8, D11, and H8) of HLA-B-engineered iPSCs were established by CRISPR gene editing with HLA-B.g2. To confirm that HLA-B.g2 induced the desired mutation, the target DNA sequence of each clone was analyzed. As shown in Fig. 2a, an insertion or deletion (Indel) was induced in each clone by HLA-B.g2. Specifically, a 1 base pair (bp) insertion was identified in clones D8 and H8, and 2 bp and 80 bp deletions were identified in clones D11 and H8, respectively. In clone H8, both an insertion and deletion were induced by HLA-B.g2. The electrophenogram of each clone is shown in Supplementary Figure S1. Compared with the wild-type control, the mutations in clones D8, D11, and H8 were predicted to cause early termination of protein translation (Supplementary Figure S2). However, the actual reduction of HLA-B expression at the mRNA and protein levels was only observed in clone H8 (Fig. 2b, c). When treated with human recombinant interferon-gamma (hrIFN-γ), the wild-type iPSC exhibited an increase in the number of HLA-B-positive cells (9.10–34.7%) in comparison with negative controls not treated with hrIFN-γ, whereas clone H8 had very few HLA-B-positive cells (4.01–7.10%), (Fig. 2d). To confirm whether the HLA-B knockout is maintained after differentiation, CRISPR-engineered iPSCs (clone H8) were first differentiated into MSCs, a widely used type of stem cell. By flow cytometry analysis, the iMSCs derived from the HLA-B-engineered iPSCs showed a similar expression level of CD73 and CD105 to that from bone marrow-derived MSCs (bMSCs) (Supplementary Figure S4). Next, the HLA-B-positive population before and after IFN-γ induction was compared between HLA-B-engineered iPSC-derived MSCs and the bMSCs, which are used for cell therapy. In Fig. 2e, bMSCs showed an increase in the HLA-B-positive population by IFN-γ induction (from 22.9 to 37.9%), whereas the HLA-B-engineered iPSC-derived MSCs showed little difference (2.02–3.98%). To confirm that HLA-B was functionally knocked out by HLA-B.g2, we performed an HLA typing assay on each clone. The HLA-A allelic types of the three clones were the same as the wild type (*24:02/*24:02), whereas the HLA-B (*40(60)/*54) allelic types of the clones were not determined (Fig. 2f). The electrophenogram of HLA-B allelic typing for each clone is provided in Supplementary Figure S3. As a further confirmation of HLA-B knockout over a long period of time, HLA typing was performed with late passage HLA-B-engineered iPSCs (50–60 passages). HLA-B (*40/*54) was identified in the wild-type control, whereas it was not in the late passage CRISPR-engineered iPSCs (D8, D11, and H8), which were matched to that of the early passage cells (data not shown). Next, to determine whether HLA-B-engineering affects the immunocompatibility of the clones, we compared the immunogenicity of each clone using HLA-targeted complement-dependent cytotoxicity assays. In the presence of HLA-B (*B60) or (*B54) antibody, each engineered clone exhibited less complement-dependent cytotoxicity (black-colored and spot-shaped cell lysis) than that in the wild-type controls (Fig. 2g). On the other hand, in the presence of HLA-A (*A24), the engineered clones exhibited a level of cytotoxicity similar to that of the wild-type control. To examine whether HLA-B-engineering (knockout) is associated with the immune-compatibility of iPSCs, an antibody-dependent cellular cytotoxicity (ADCC) assay was performed in the HLA-B-engineered iPSC-derived MSCs and bMSCs. The bMSCs showed a similar level of cell proliferation in the presence of anti-HLA-B antibody to the control, but the level of cell proliferation was decreased in the presence of anti-HLA-B antibody and rabbit complement. In contrast, HLA-B-engineered iPSC-derived MSCs showed an increase in cell proliferation in the presence of anti-HLA-B antibody and/or rabbit complement (Fig. 2h).

### Characterization of HLA-B-engineered iPSC clones

**Fig. 3:**
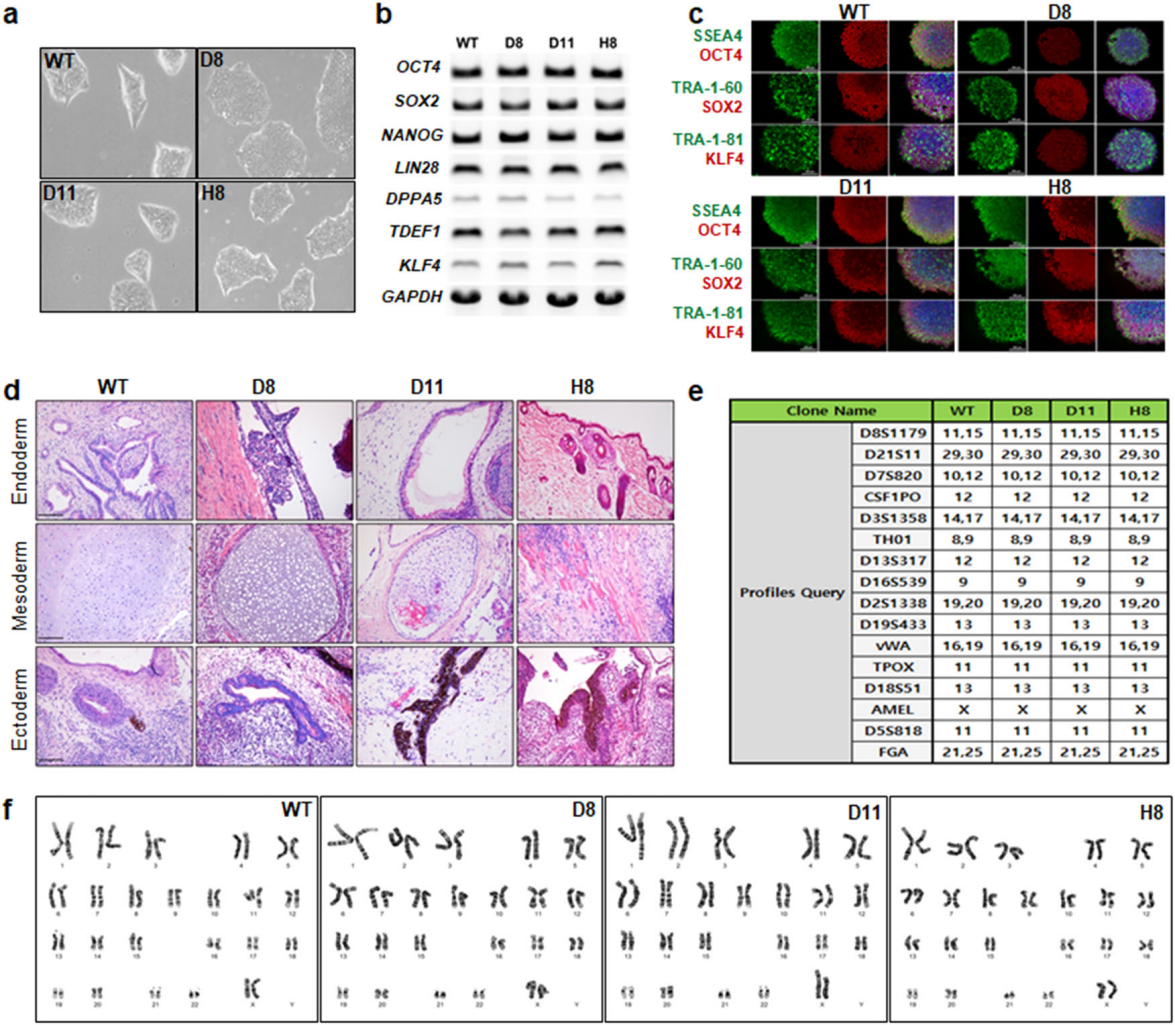
Characterization of HLA-B–engineered iPSC clones. **a** Representative morphologies of HLA-B-engineered iPSC clones. Images were acquired on a Leica microscope (Leica Microsystems Ltd, EC3, image 40 × ). Expression analysis of stemness markers in the wild-type and HLA-B-engineered iPSC clones was performed at the **b** mRNA and **c** protein levels by RT-PCR and immunofluorescence staining, respectively (scale bar, 200 μm). **d** Teratoma assay with wild-type and HLA-B-engineered iPSCs (scale bar, 200 μm). **e** Genetic profiling of wild-type and HLA-B-engineered iPSC clones. STR was based on the multiplex analysis of 15 loci and the amelogenin gender-determination marker. **f** Representative karyotypes of wild-type and HLA-B-engineered iPSC clones

To characterize the HLA-B-engineered iPSC clones, we compared their growth morphologies to those of wild-type iPSCs by microscopy; this analysis revealed no significant morphological difference (Fig. 3a). To determine whether CRISPR-derived engineering of HLA-B affects the stemness of iPSC clones, we performed RT-PCR to measure the expression of mRNAs encoding a set of stemness markers (*OCT4*, *SOX2*, *NANOG*, *LIN28*, *DPPA5*, *TDEF1*, and *KLF4*). At the protein level, we performed immunofluorescence staining with antibodies against SSEA4, OCT4, TRA1–60, SOX2, TRA-1–81, and KLF4. As shown in Fig. 3b and c, there was little difference in the expression of stemness markers, at the mRNA and protein level, between the clones and wild-type cells. To confirm that HLA-B-engineered (KO) iPSCs maintain stemness over a long period, we determined the expression of stem cell markers (SSEA4, OCT4, TRA-160, SOX2, TRA-1–81, and KLF4) and found little difference between the wild-type iPSCs, early passage (30–40 passage), and late passage (50–60) HLA-B-engineered iPSCs (data not shown). To confirm whether the genetic editing of HLA-B affects the pluripotency of stem cells, we performed a teratoma formation assay of HLA-B-engineered iPSCs and observed a similar level of spontaneous differentiation potential in the in vivo model (NOD, CB17-Prkdcscid/J) transplanted with wild-type and HLA-B-engineered iPSCs. The commitment of the iPSC line to all three germ layers was confirmed by hematoxylin and eosin staining in which endodermal, mesodermal and ectodermal differentiation were detected. Like wild-type iPSCs, HLA-B-engineered iPSCs showed a similar level of ectodermal, endodermal, and mesodermal differentiation (Fig. 3d). To investigate the possibility that HLA-B engineering altered the characteristics of wild-type iPSCs, we confirmed the genetic identity of each HLA-B-engineered iPSC clone by short tandem repeat (STR) and karyotype analyses. Each clone showed the same STR profile as the wild-type clones (Fig. 3e). In addition, all clones showed the same karyotypes as the wild type (Fig. 3f).

### Analysis of differentiation capacity of HLA-B-engineered iPSC clones

**Fig. 4:**
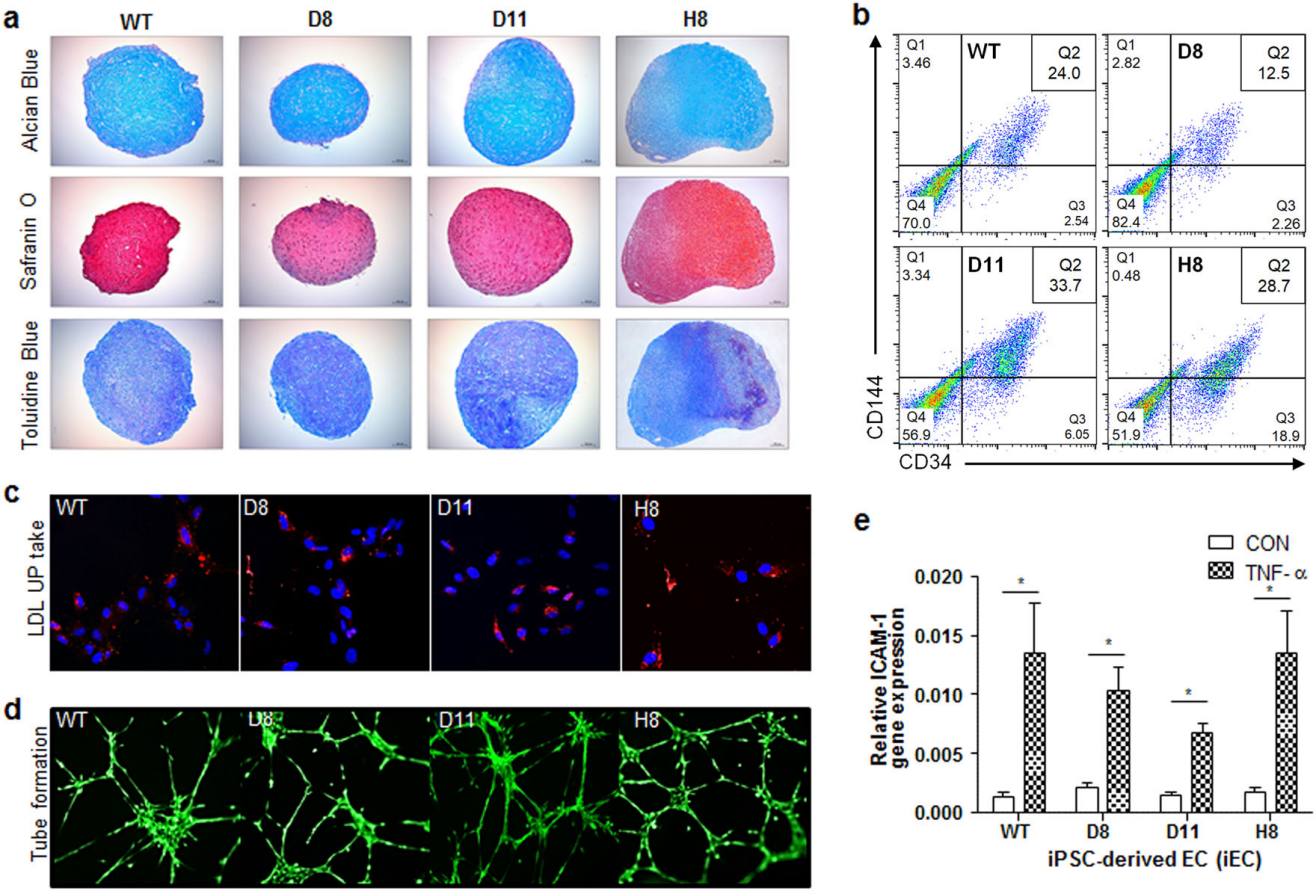
Differentiation capacity analysis of HLA-B–engineered iPSC clones. Chondrogenic differentiation analysis of wild-type and HLA-B-engineered iPSC clones. Glycosaminoglycan, **a** major extracellular matrix in cartilage tissue, was stained with Alcian blue (Blue), Safranin O (Orange to red), and Toluidine Blue (Purple). Images were acquired on a Leica microscope (DM50003; scale bar, 100 μm). **b** Endothelial cell differentiation of wild-type and HLA-B-engineered iPSC clones. The CD34- or CD144-positive population was evaluated in each clone by flow cytometry. **c** Florescent LDL uptake assay and **d** tube formation assay of the iECs derived from wild-type and HLA-B-engineered iPSCs. **e** TNF-α-induced expression of ICAM-1 in the iECs derived from wild-type and HLA-B-engineered iPSCs. One asterisk indicates a *p-*value less than 0.05

Next, we monitored the in vitro chondrogenic and endothelial cell differentiation to determine whether HLA-B engineering affected the differentiation capacity of iPSC clones. In particular, chondrogenic differentiation was performed using three-dimensional (3D) spheroid culture. On day 30, the differentiation phenotype was confirmed in chondrogenic spheroid pellets of HLA-B-engineered iPSC clones by Alcian blue, Safranin O, and Toluidine Blue staining. The chondrogenic differentiation capacity of the HLA-B-engineered iPSC clones was barely different from that of the wild-type clones. Specifically, the chondrogenic pellets of HLA-B-engineered iPSC clones exhibited a cartilage-like pattern of staining similar to that of the wild type (Fig. 4a). Endothelial cell differentiation was also induced in the HLA-B-engineered iPSC clones. On day 8, the expression of endothelial cell markers (CD34 and CD144) was evaluated by flow cytometric analysis. In Fig. 4b, the CD34/CD144 double-positive population was 24% in the wild type, 12.5% in clone D8, 33.7% in clone D11, and 28.7% in clone H8. In addition, to confirm variation among iPSC colonies, we analyzed the ability of an individual colony in each clone to undergo endothelial cell differentiation. For this purpose, we selected three colonies each from the wild-type and HLA-B-mutated iPSC clones (D8, D11, and H8). We observed no significant difference among the endothelial cell differentiation potentials of each iPSC clone. A histogram analysis of each colony from each HLA-B-engineered and wild-type clone is provided in Supplementary Figure S4. To confirm whether HLA-B gene editing caused the loss of EC function, low-density lipoprotein (LDL) metabolism, which is known to be high in ECs^36^, was measured in the iECs differentiated from the parental and HLA-B-engineered iPSCs using acetylated low-density lipoprotein labeled with Dil (Dil-Ac-LDL) incorporation. In Fig. 4c, there was no significant difference in Dil-Ac-LDL incorporation between the wild-type and HLA-B-engineered iECs. In the tube formation assay to evaluate angiogenic potential^37^, the clones from the iECs differentiated from the parental and HLA-B-engineered iPSCs showed little difference as shown in Fig. 4d. ICAM-1 is upregulated by TNF-α in ECs^38^. Therefore, the TNF-α-induced expression of ICAM-1 was compared in the iEC clones from the wild-type and HLA-B-engineered iPSCs. In Fig. 4e, both iEC clones showed a similar pattern of ICAM-1 expression.

## Discussion

Genetic engineering of HLA alleles was proposed recently as a means of developing immunocompatible donor stem cells^39,40^. However, whether HLA-engineered stem cells are suitable for allotransplantation has not been demonstrated. In this study, as a proof of concept, we induced the functional knockout of HLA-B alleles from iPSCs using the CRISPR/Cas9 system, and then tested the immunocompatibility of the engineered cells using a complement-dependent cytotoxicity assay.

Based on the DNA sequencing analysis, HLA-B (*40:01(60) and *54:01) was functionally knocked out in all three clones due to the early termination of translation (Supplementary Figure S2), theoretically resulting in the reduced expression of HLA-B. According to the IPD-IMGT/HLA database, the gRNA HLA-B.g2 showed a perfect match to the HLA-B*40 allele, whereas it had three mismatches to the HLA-B*54 allele. Therefore, the expression HLA-B*54 was highly likely to be maintained in the CRISPR-edited iPSCs. In general, a 3 bp mismatched gRNA is known to be nonfunctional^41^. Interestingly, however, a predominant decrease in HLA-B expression was observed in clone H8, but not in clones D8 and D11, at both the mRNA and protein levels (Fig. 2b, c). Unlike the analysis of HLA-B expression, the complement-dependent cytotoxicity (CDC) with anti-HLA-B (B*60 and B*54) antibodies was clearly lower in all three clones than that in the wild type (Fig. 2g). Compared with bMSCs, a widely used type of stem cells for cell therapy, the CDC with anti-HLA-B was dramatically lower in HLA-B-engineered iPSC-derived MSCs (Fig. 2h). The discrepant results of mRNA and protein expression between the CRISPR-engineered iPSC clones may be caused by the high sequence similarity of the HLA molecules. Unfortunately, an antibody specifically distinguishing HLA-B molecules from others has not been commercially developed. Therefore, an antibody against MHC class 1 was used in several studies instead of an anti-HLA-B antibody^22,42,43^.

The most encouraging point of this study is that HLA-B-engineered iPSC clones maintained levels of stemness and differentiation capacity similar to those of the wild-type clones. All three clones could be differentiated into chondrocytes and endothelial cells as efficiently as wild-type cells (Fig. 4a–e), indicating that CRISPR-based engineering of HLA in iPSCs is a promising approach for developing immunocompatible and ready-to-use donor stem cells.

In summary, we investigated two important aspects of a recently proposed strategy for the development of donor stem cells: the immunocompatibility and differentiation capacity of HLA-engineered iPSCs generated by the CRISPR/Cas9 system. Based on our results, we suggest that CRISPR-based engineering of HLA is a good approach for developing immunocompatible and ready-to-use donor stem cells.

## Electronic supplementary material

Supplemental information

Supplemental Figure S1

Supplemental Figure S2

Supplemental Figure S3

Supplemental Figure S4
