# Supplementary material for: Development of immunocompatible pluripotent stem cells via CRISPR-based human leukocyte antigen engineering
Source: Exp Mol Med. 2019 Jan 7;51(1):3. doi: 10.1038/s12276-018-0190-2 (PMC6323054; doi:10.1038/s12276-018-0190-2)
Supplement: Supplementary file 1 — Supplemental information [file 12276_2018_190_MOESM1_ESM.docx]

**Supplemental Information**

**Supplemental Figure legends**

**Supplemental Figure S1. Electrophenogram analysis of CRISPR-engineered HLA-B clonal sequences**

Nucleotide sequence analysis of HLA-B–engineered hiPSC clones. The gRNA HLA-B.g2 was transfected into hiPSCs and then single-cell cloning was performed by puromycin selection. Three HLA-B–mutated clones (D8, D11, and H8) were established. Genomic DNA was extracted from the wild type and each HLA-B– engineered clone, and PCR was performed. PCR products were subjected to Sanger sequencing to identify the indels generated by NHEJ events resulting from DSBs in the HLA-B locus. The genetic modifications in the HLA-B sequence were identified as a 1 nt insertion, 2 nt deletion, and 80 nt deletion with a 1 nt insertion in D8, D11, and H8, respectively.

**Supplemental Figure S2. Open reading frame analysis of CRISPR-engineered HLA-B clones**

Protein translation analysis of CRISPR-engineered nucleotide sequences. The predicted protein sequence of each clone is compared to that of wild-type hiPSCs. The analysis was performed using an online DNA-to-protein translation tool, translator (www.fr33.net/translator.php).

**Supplemental Figure S3. Electrophenogram analysis of HLA allelic typing for HLA-B–engineered iPSC clones**

HLA-B type analysis of the wild-type clone was confirmed using a specific primer. HLA-B type analysis of HLA-B-engineered clones could not be performed because the specific primer did not bind to the DNA of HLA-B-engineered clones D8, D11, and H8.

**Supplemental Figure S4. Histogram analysis of the CD34- and CD144-positive populations of HLA-B–engineered iPSC clones in which epithelial cell differentiation was induced**

a) Comparison of the endothelial cell differentiation potential in three colonies of each iPSC clone. Three colonies each of the wild type and HLA-B-engineered iPSC clones D8, D11, and H8 were selected for this experiment. The ability of each colony to undergo endothelial cell differentiation was analyzed to confirm variation among iPSC colonies. Expression of two endothelial cell markers, CD34 and CD144, was confirmed by flow cytometry. Data were collected from 20,000 events. b) MSC marker expression of HLA-B–engineered iPSC-derived MSC (iMSC). bMSCs were used as a positive control.

**Supplemental Tables**

**Supplemental Table S1. Off-target analysis for the gRNA to generate HLA-B KO clone**

| Name | gRNA | Long_0 | Long_1 | Long_2 |
| --- | --- | --- | --- | --- |
| MS540.HLA-B.g54 | CGTCGCAGCCGTACATGCTCNGG | 1 | 1 | 1 |
| MS540.HLA-B.g3 | AGCATGTACGGCTGCGACGTNGG | 1 | 1 | 1 |
| MS540.HLA-B.g2 | GAGCATGTACGGCTGCGACGNGG | 1 | 1 | 1 |
| MS540.HLA-B.g50 | AGGCGTACTGGTCATGCCCGNGG | 1 | 1 | 2 |

Long_0: Number of site in genome that are an exact match to “long’, including target site

Long_1: Number of site in genome that contain up to 1 mismatch in “long’, including target site

Long_2: Number of site in genome that contain up to 2 mismatch in “long’, including target site

**Supplemental Table S2. Primer information**

| Primer | Sequence (5'→3') | Product size (bp) |
| --- | --- | --- |
| *OCT4* | ACCCCTGGTGCCGTGAA | 190 |
|  | GGCTGAATACCTTCCCAAATA |  |
| *SOX2* | CAGCGCATGGACAGTTAC | 321 |
|  | GGAGTGGGAGGAAGAGGT |  |
| *NANOG* | AAAGGCAAACAACCCACT | 270 |
|  | GCTATTCTTCGGCCAGTT |  |
| *LIN28* | GTTCGGCTTCCTGTCCAT | 122 |
|  | CTGCCTCACCCTCCTTCA |  |
| *DPPA5* | CGGCTGCTGAAAGCCATTTT | 215 |
|  | AGTTTGAGCATCCCTCGCTC |  |
| *TDEF1* | TCCTTCTACGGACGGAACTG | 140 |
|  | AGAAATGCCTGAGGAAAGCA |  |
| *KLF4* | CAGTGCCAAAAATGCGACCGAGC | 188 |
|  | GACCATGATTGTAGTGCTTTCTGGC |  |
| *GAPDH* | GAATGGGCAGCCGTTAGGAA | 414 |
|  | GACTCCACGACGTACTCAGC |  |
| *HLA-B* | AGGCGCGTTTACCCGGTTTC | 339 |
|  | GAGCCACTCCACGCACTC |  |
| *ICAM-1* | CAAGGCCTCAGTCAGTGTGA | 149 |
|  | CCTCTGGCTTCGTCAGAATC |  |

**Supplemental Table S3. Antibody information**

| Antibody | Manufacturer | Dilution ratio |
| --- | --- | --- |
| SSEA4 | EMD Millipore | 1/200 |
| OCT | Santa Cruz Biotechnology, Dallas, TX | 1/100 |
| TRA-1-60 | EMD Millipore | 1/100 |
| SOX2 | BioLegend, San Diego, CA | 1/100 |
| TRA-1-81 | EMD Millipore | 1/100 |
| KLF4 | Abcam, Milton, Cambridge, UK | 1/250 |
| HLA-A | Abcam | 1/1000 |
| HLA-B | Thermo scientific, Rockford, IL  Abcam | WB;1/1000 |
|  |  | FACS: 1/50 |
|  |  | ADCC:1/200 |
| GAPDH | Thermo scientific | 1/5000 |
| CD34-APC | eBioscience, San Diego, CA | 0.25 μg/test |
| CD73-PE | eBioscience | 0.5 μg/test |
| CD105- PE-cyanine7 | eBioscience | 0.5 μg/test |
| CD144-PE-cyanine7 | eBioscience | 0.5 μg/test |
| Mouse IgG1 kappa Isotype Control, APC | eBioscience | 0.25 μg/test |
| Mouse IgG1 kappa Isotype Control, PE-cyanine7 | eBioscience | 0.5 μg/test |

**Supplemental Table S4. HLA allele analysis of the cord blood bank**

| Gene | *HLA A* | *HLA B* | | *DR* | |
| --- | --- | --- | --- | --- | --- |
| Allele | 1 & 2 | 1 | 2 | 1 | 2 |
|  | *33:03:01 | *14 | *44 | - ^a^ | - |
|  |  | *44 | *58 | - | - |
|  |  | *44 | | *07 | *13 |
|  |  | *44 | | *07 | *13 |
|  |  | *44 | | *07 | *13 |
|  |  | *44 | | *01 | *13 |
|  | *24:02:01:01 | *40:01(60) | *54 | - | - |
|  |  | *40:01(61) | *51 | - | - |
|  |  | *54 | *15:18(71) | - | - |
|  |  | *40:02(61) | *51 | - | - |
|  |  | *51 | *54 | - | - |
|  |  | *40:02(61) | *54 | - | - |
|  |  | *52 | *15:01(62) | - | - |
|  |  | *35 | *59 | - | - |
|  |  | *40:02(61) | *51 | - | - |
|  |  | *35 | *59 | - | - |
|  | *11:01:01 | *35 | *15:01(62) | - | - |
|  |  | *13 | *27 | - | - |
|  |  | *54 | *57 | - | - |
|  |  | *35 | *15:01(62) | - | - |
|  |  | *35 | *15:01(62) | - | - |
|  | *31:01:02 | *48 | *51 | - | - |
|  | *02:01:01:01 | *51 | *15:11(75) | - | - |
| Homozygous Allele | 23 Cases | 4 Cases | 0 Cases | 0 Cases |  |

N=127.

^a^Not typed.
