# Supplementary figures and images for: Development of immunocompatible pluripotent stem cells via CRISPR-based human leukocyte antigen engineering
Source: Exp Mol Med. 2019 Jan 7;51(1):3. doi: 10.1038/s12276-018-0190-2 (PMC6323054; doi:10.1038/s12276-018-0190-2)

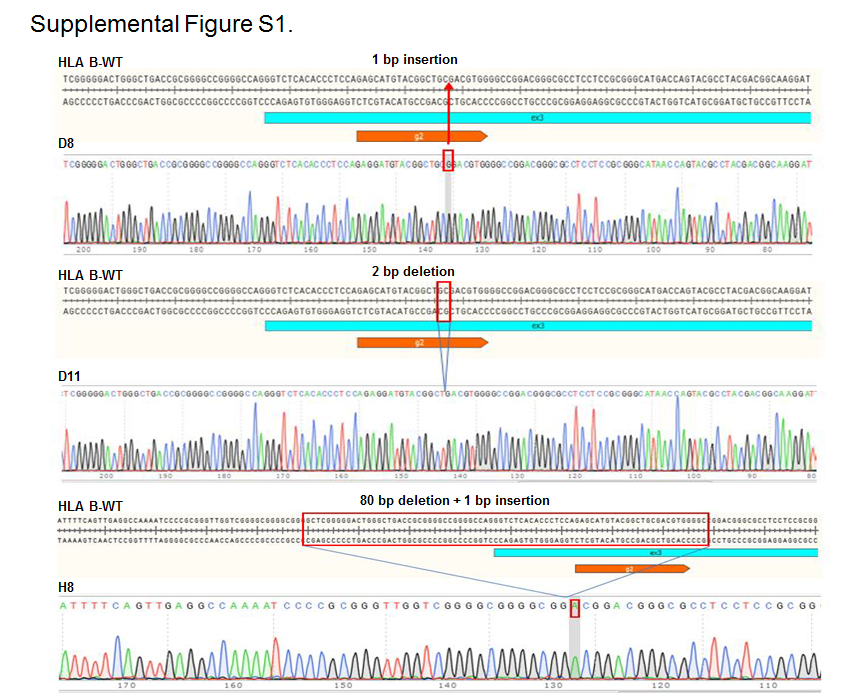

Supplement: Supplementary file 2 — Supplemental Figure S1 [file 12276_2018_190_MOESM2_ESM.tif]

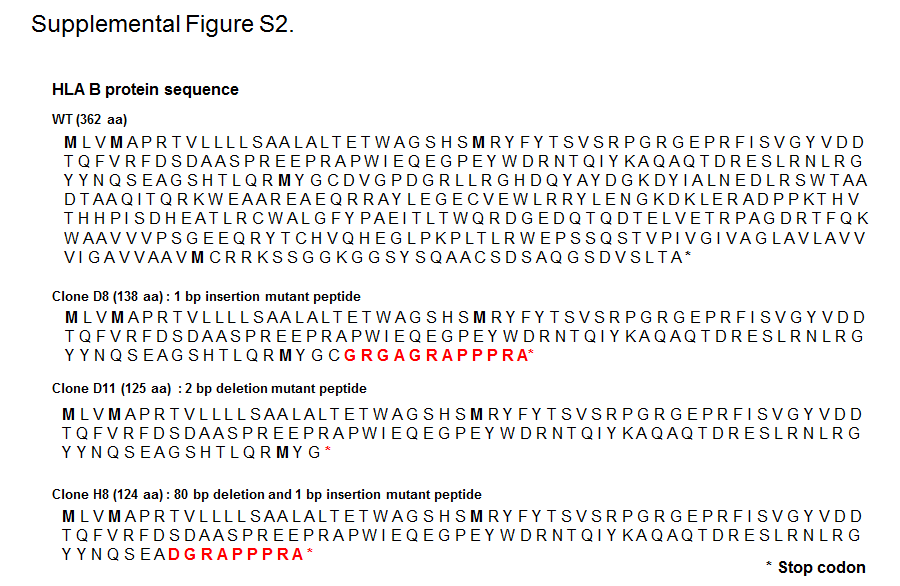

Supplement: Supplementary file 3 — Supplemental Figure S2 [file 12276_2018_190_MOESM3_ESM.tif]

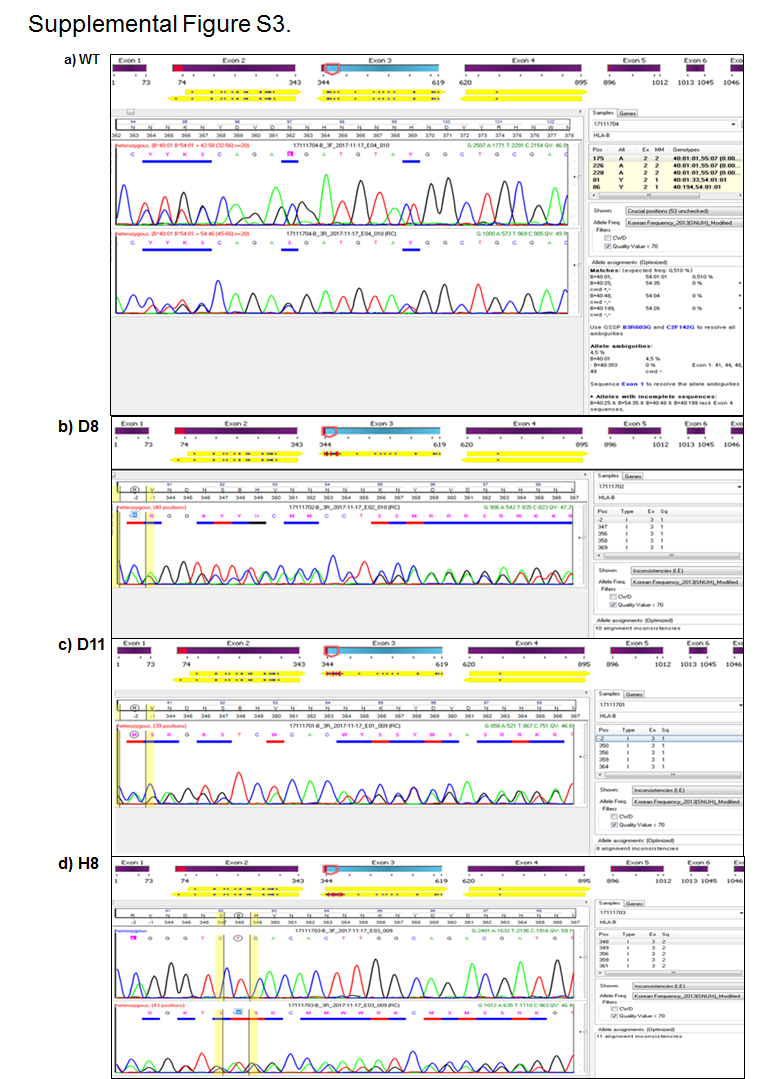

Supplement: Supplementary file 4 — Supplemental Figure S3 [file 12276_2018_190_MOESM4_ESM.tif]

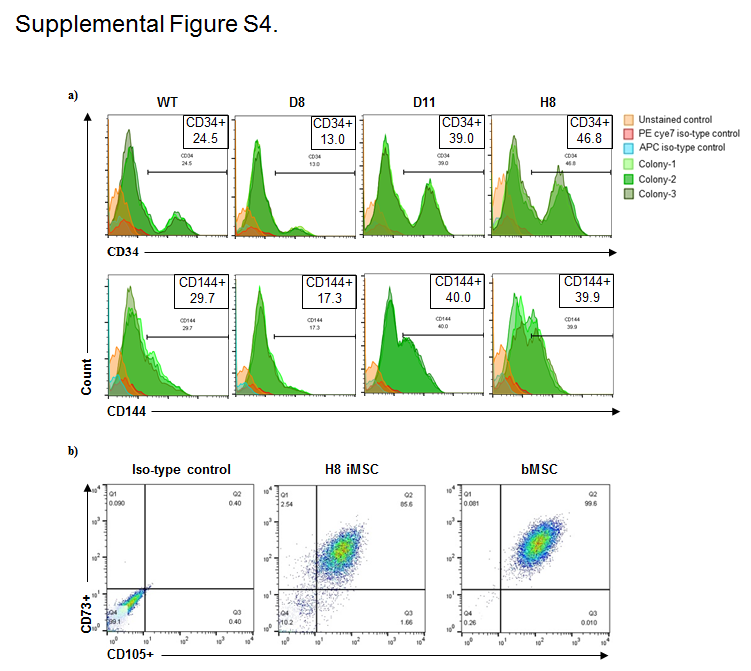

Supplement: Supplementary file 5 — Supplemental Figure S4 [file 12276_2018_190_MOESM5_ESM.tif]
